# Core Needle Biopsy Can Early and Precisely Identify Large Thyroid Masses

**DOI:** 10.3389/fonc.2022.854755

**Published:** 2022-04-05

**Authors:** Antonio Matrone, Luigi De Napoli, Liborio Torregrossa, Aleksandr Aghababyan, Piermarco Papini, Carlo Enrico Ambrosini, Rosa Cervelli, Clara Ugolini, Fulvio Basolo, Eleonora Molinaro, Rossella Elisei, Gabriele Materazzi

**Affiliations:** ^1^ Department of Clinical and Experimental Medicine, Unit of Endocrinology, University Hospital of Pisa, Pisa, Italy; ^2^ Department of Surgical, Medical, Molecular Pathology and Critical Area, Unit of Endocrine Surgery, University Hospital of Pisa, Pisa, Italy; ^3^ Department of Surgical, Medical, Molecular Pathology and Critical Area, Anatomic Pathology Section, University Hospital of Pisa, Pisa, Italy; ^4^ Division of Interventional Radiology, University Hospital of Pisa, Pisa, Italy

**Keywords:** anaplastic thyroid carcinoma, poorly differentiated thyroid carcinoma, core needle biopsy, fine needle aspiration cytology, thyroid lymphoma

## Abstract

**Background:**

Large thyroid masses, particularly if rapidly growing, are often characterized by compression and infiltration of the vital structures of the neck. Therefore, an early and precise diagnosis, not only of malignancy but also of histotype, is mandatory to set up the right therapy. The aim of this study was to evaluate the diagnostic performance of fine needle aspiration cytology (FNAC) and core needle biopsy (CNB) in this setting.

**Patients and Methods:**

We prospectively evaluated 95 patients with large and rapidly growing thyroid masses admitted to the University Hospital of Pisa between April 2014 and January 2020. All patients were submitted to FNAC and CNB in the same session. The ability of both procedures to diagnose the malignancy of the lesions, particularly the histotype, and to obtain sufficient material to perform molecular analysis was evaluated.

**Results:**

FNAC obtained adequate tumor sample to reach a diagnosis in 76 of 95 (80%) patients, while a higher percentage was obtained with CNB (92/95, 96.8%). FNAC was able to identify the malignancy of the lesion in 74 of 95 (77.9%) cases, but only in 16 of 74 (21.6%) cases was it able to define the histotype. CNB was able to define the malignancy of the lesion in all but three cases (92/95, 96.8%), and in all specimens, the histotype was identified. Moreover, in all cases, the material extracted from CNB was optimal to perform molecular analysis. No surgery-related complications were experienced with both procedures.

**Conclusions:**

CNB is a rapid and safe procedure with higher performance compared to FNAC in identifying the histotype of large and rapidly growing thyroid masses. Moreover, adequate material can be obtained to characterize the molecular profile for the treatment of potentially lethal cancers. In the era of precision medicine, CNB should be introduced in routine clinical practice as a key procedure for an early diagnosis and therapy of these diseases.

## Introduction

Large thyroid masses, particularly if rapidly growing, are often life-threatening events because of the mechanical compression and/or infiltration of the vital structures of the neck (1–3). The most common is anaplastic thyroid carcinoma (ATC), which often appears with hoarseness, cervical pain, dysphagia, dyspnea, and stridor (4, 5). Moreover, beyond local compression, ATC is characterized by high rates of distant metastases and rapidly fatal clinical outcomes (6), and most patients die within 6 months from the diagnosis (1, 7). Conversely, large thyroid masses other than ATC are not necessarily life threatening and can be successfully treated with specific therapies. A clear identification of the histology of these masses is essential to select the most appropriate therapeutic approach (8–10).

All ATC cases are defined as stage IV and primary tumor considered T4 by the American Joint Committee on Cancer (11). According to the American Thyroid Association (12), the therapeutic options include radical surgery, if possible, external radiotherapy, and/or chemotherapy, which should be combined to maximize the disease control (3, 13, 14).

Since ATC is highly aggressive, a rapid diagnosis and treatment should be mandatory. Moreover, since other thyroid masses can have similar clinical presentation, but different outcomes, differentiating ATC from thyroid lymphoma (TL), poorly differentiated thyroid cancer (PDTC), and thyroid gland metastases (TGM) could improve the therapeutic approach and survival (8).

Currently, fine needle aspiration cytology (FNAC) is the most common diagnostic procedure (3), but it has shown several limitations particularly in the identification of ATC or TL (15–17). Therefore, patients with a suspicion of ATC or TL frequently require a surgical conventional biopsy, with more time elapsed to achieve the correct diagnosis (18). Moreover, in the case of surgical conventional biopsy, particularly in ATC, the surgical wound does not quickly heal, thus leading to a further delay in beginning potential treatments.

Core needle biopsy (CNB) represents a minimally invasive, safe, and accurate procedure providing a histological sample, which retains not only its cytologic appearance but also the tissue architecture. CNB has been suggested as a complementary method to FNAC, mainly to overcome possible inconclusive diagnosis (19). Accordingly, FNAC can play an important diagnostic role in the initial evaluation of ATC, but CNB may be necessary for definitive diagnosis and to perform molecular analysis (3). To our knowledge, no studies comparing the sensitivity of FNAC and CNB, on the same patient, in the diagnostic accuracy for detecting the malignancy of large thyroid masses and discriminating the histology have been performed.

The aim of this prospective study was to compare the diagnostic performance of FNAC and CNB in a large series of patients, thus exploring the possibility that CNB could be the first and main diagnostic tool in the presence of a large and rapidly growing thyroid mass.

## Patients and Methods

We prospectively collected the data of 95 consecutive patients with large thyroid masses admitted to either the Endocrine Unit or the Endocrine Surgery Unit of the University Hospital of Pisa between April 2014 and January 2020.

The study was conducted according to the guidelines of the Declaration of Helsinki, approved by the local Ethical Committee (CEAVNO, Comitato Etico Area Vasta Nord Ovest). For the policy of the University Hospital, all patients signed an informed consent both for the performance of invasive procedures and for the use of their data for scientific purposes.

### Preparation for FNAC and CNB

All patients were submitted to routine blood evaluation and coagulation tests, and the medical history and hemorrhagic risk were carefully evaluated. When receiving antiplatelet or anticoagulation therapy, the diagnostic procedures were still performed, without withdrawal. Only warfarin was discontinued up to 3 days before the procedure. No antibiotics and/or analgesics were used after the procedures.

For each patient, a total body spiral computed tomography (CT) scan with intravenous contrast medium, particularly focused on the neck–mediastinum, was performed. Bronchoscopy and esophagoscopy were performed, if needed.

As per protocol, all patients were submitted to FNAC and CNB in the same time session.

### Neck Ultrasound and CT Scan

An Aloka ProSound Alpha-5sv ultrasound system with a 7.5- to 12-MHz linear transducer (Hitachi Aloka Medical, LTD., Tokyo, Japan) was used for the neck ultrasound (neck US) examination. Neck US assessment is necessary to evaluate the composition and vascularity of the lesion in order to avoid necrotic spaces and vascular bundle and to select the most appropriate area of the mass for tissue sampling. For both procedures, a trans-isthmic or a lateral approach was performed according to each specific case.

A Lightspeed 16 RT, Lightspeed 64 VCT, and a Discovery HD 750 CT scan (GE Medical Systems, Waukesha, WI, USA) was used in patients scanned in our institution. Images of the total body CT scan were utilized for the evaluation of the tumor dimension; the presence of necrosis and/or calcifications; esophageal, tracheal, or laryngeal invasion; vascular involvement; and lymph node and/or distant metastases.

### FNAC Procedure

Local anesthesia with 1% lidocaine was applied just before performing both FNAC and CNB, and a 2- to 3-mm skin incision was performed using no. 11 surgical blade, specifically for the aim of this study.

US-guided FNAC was performed with a 21-gauge needle using a 10- or 20-ml syringe with standard aspiration technique. During FNA, four to eight needle passes were performed during one single puncture in the analyzed nodule. The appropriateness of the material obtained by FNA was evaluated on site by the endocrinologist or the surgeon who performed the procedure and was then used to prepare smears, which were examined by pathologists after staining with hematoxylin and eosin.

Cytological results were classified based on the Italian Consensus (20). Accordingly, the samples were defined as non-diagnostic if they were “inadequate”, when biased by smearing and/or fixing and/or staining artifacts or by obscuring blood, or “non-representative”, when the number of epithelial cells collected from the mass was insufficient for a definitive diagnosis. After FNA, we immediately performed CNB using the same skin incision.

### CNB Procedure

CNB was performed using a 16-gauge disposable double-component spring-activated needle. The needle was positioned above the mass, in the same point of the previous FNAC, and was pushed to shoot the cutting cannula. The entire procedure was US guided. The biopsy needles were about 100 mm long. In all cases, we used a 2.0-cm excursion needle. Usually, two or three core samples were picked up from the same skin incision and fixed with 10% formaldehyde solution. After biopsy, the skin incision was dabbed and disinfected, but not sutured. Immediately after CNB, a manual compression of the neck was applied by the patient, and all patients were observed in hospital for the following 20–30 minutes.

Both procedures, FNAC and CNB, were conducted in Fowler’s position to avoid respiratory failure.

### Cytological and Histological Analyses

FNAC and CNB were analyzed by three different pathologists (LT, CU, and FB) in a double-blinded protocol. Immunohistochemical analyses were performed on each tissue sample obtained by CNB using the VENTANA BenchMark immunostaining system (Ventana Medical Systems, Tucson, AZ, USA). From formalin-fixed and paraffin-embedded specimens, tissue sections (3–5 mm) were deparaffinized and processed. Different panels of immunostaining were performed according to the morphological aspect on tissue section.

Since a greater amount of tissue was obtained by CNB, we decided to perform immunohistochemical and molecular analyses only on CNB samples.

### Molecular Analysis Data Collection

Molecular analysis of samples was not an aim of this study and was not performed systematically. However, we collected the available data found in the pathological report for a descriptive analysis. From 2018, genetic analysis for potential actionable mutations, such as *BRAF* V600E first and then *RET/PTC* and *NTRK* rearrangements, has been performed (21–24). Moreover, in some cases, other oncogenes, especially those beneficial for a differential diagnosis, were analyzed. Good quality DNA and RNA extracted from CNB were obtained and were used to perform molecular analysis. We then performed real-time PCR to analyze codons 600 and 601 of the *BRAF* gene (EasyPGX^®^ ready THYROID), *RET/PTC* 1–3, *NTRK* 1–3, and *PAX8/PPAR* gamma rearrangements (EasyPGX^®^ ready *NTRK* and THYROID Fusion). Analysis of the *TERT* gene promoter hotspot mutations C228T and C250T and the *TP53* gene mutations in exons 4–9 was performed on specific request, not in all cases, using direct Sanger sequencing. The MassARRAY system (Sequenom, San Diego, CA, USA) was utilized for the evaluation of exons 18–21 of the *EGFR* gene, exon 20 of the *HER-2* gene, and exons 9 and 20 of the *PIK3CA* gene. Fluorescence *in situ* hybridization (FISH) analysis was conducted for *ROS1* rearrangement (Vysis 6q22 ROS1 Break Apart FISH Probe Kit), MYC translocation (Vysis MYC Break Apart FISH Probe Kit), and BCL2 translocation (Vysis BCL2 Break Apart FISH Probe Kit (all from Abbott Laboratories, Chicago, IL, USA).

### Statistical Analysis

Data are presented as median and interquartile range (IQR) or as frequency (percentage). Diagnostic accuracy was evaluated for both procedures. The *χ* ^2^ test was used to assess differences between the categorical variables in both procedures. A *p*-value <0.05 was considered statistically significant. Statistical analysis was performed with SPSS 21.0 software (IBM Corp., Armonk, NY, USA).

## Results

At the time of enrollment, patients had a median age of 70 years (IQR = 58–79 years, range = 28–91 years), and 55 out of 95 (57.9%) were women (**Table 1**).

**Table 1:** Epidemiological features of the study group (*n* = 95) and CT scan features of the 76 patients (76/95, 80%) with large thyroid masses who had a CT scan in our department.

Features			*n* (%)
Sex		M	40 (42.1)
		F	55 (57.9)
Age (years)		Median = 70 years (IQR = 58–79, range = 28–91)	
Volume (ml) (*n* = 76, 80%)		Thyroid gland including surrounding parenchyma	114.5
		Malignant lesion alone	106.5
CT scan features (*n* = 76, 80%)	Infiltration	Absent	20 (26.3)
		Esophagus	3 (3.9)
		Trachea	4 (5.3)
		Vascular	12 (15.8)
		Esophagus and trachea	11 (14.5)
		Esophagus and vascular	2 (2.6)
		Trachea and vascular	8 (10.5)
		Esophagus, trachea, and vascular	16 (21.1)
	Calcifications	Absent	38 (50)
		Micro	16 (21.1)
		Macro	22 (28.9)
	Necrosis	Absent	28 (36.8)
		Present	48 (63.2)
	Lymph node metastases	Absent	15 (19.7)
		Present	61 (80.3)
	Distant metastases	Absent	33 (43.4)
		Present	42 (55.3)
	Site of distant metastases	Lung	33 (78.7)
		Liver	3 (7.1)
		Bone	–
		Lung and liver	3 (7.1)
		Other	3 (7.1)

### CT Scan Results

In 19 out of 95 (20%) cases, CT scan was not performed in our hospital, and images were evaluated at the time of procedures by the medical team involved in the treatment of the patients. Conversely, 76 of 95 (80%) patients had the CT scan done in our hospital, and images were reviewed by a dedicated radiologist (RC); the radiological features of the neck mass of these patients are reported in **Table 1**.

The median estimated volume of the thyroid gland, including the surrounding parenchyma not involved in the neoplasia, was 114.5 ml (IQR = 48.25–232.5), while that of the malignant lesion was 106.5 ml (IQR = 40.5–210.75). In most cases, the CT scan showed the invasion of some structures of the neck (73.7%), and about half of them revealed the presence of intratumoral macro- or microcalcifications (50%) and necrosis (63.2%). Lymph node (80.3%) and distant (55.3%) metastases were already present at diagnosis.

**Figure 1:**
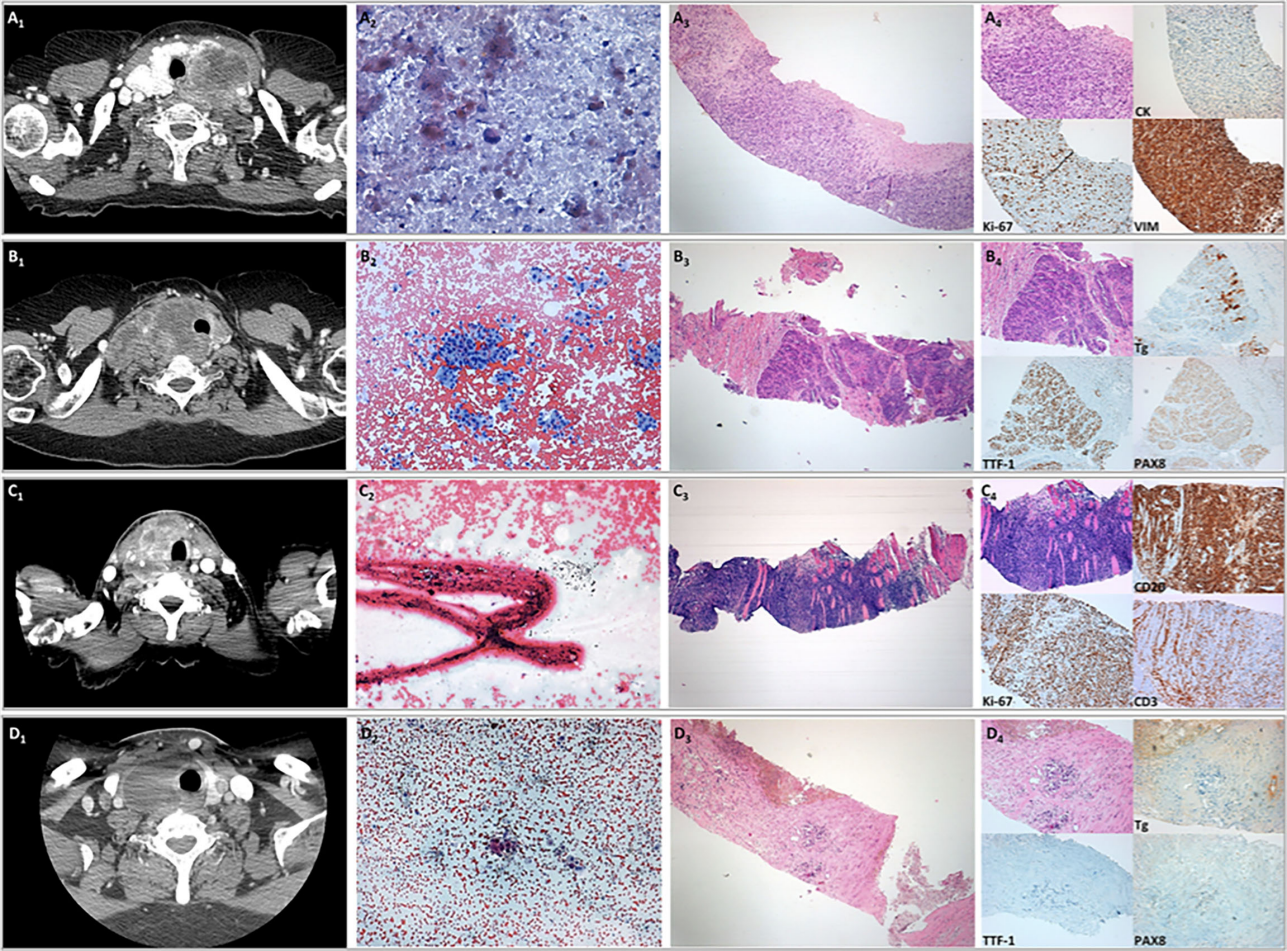
Correlation of the results of CT scan with those of fine needle aspiration cytology (FNAC), core needle biopsy (CNB), and specific immunohistochemical staining for anaplastic thyroid carcinoma (ATC), poorly differentiated thyroid cancer (PDTC), thyroid lymphoma (TL), and thyroid gland metastases (TGM) of lung adenocarcinoma. **(A)** Representative cytological and histological images of a case of ATC. (*A1*) CT scan with i.v contrast imaging. (*A2*) FNAC sample showing a few isolated atypical cells in a necrotic background (original magnification, ×40; Papanicolaou staining). (*A3*) CNB provided a tissue fragment composed of malignant undifferentiated neoplasia (original magnifications, ×4 and ×10; H&E staining). (*A4*) Immunohistochemical staining showing neoplastic cells with a high proliferative index, immunoreactivity for vimentin, and patchy weak immunopositivity for cytokeratins. **(B)** Representative cytological and histological images of a case of poorly differentiated thyroid carcinoma. (*B1*) CT scan with i.v contrast imaging. (*B2*) FNAC sample showing numerous groups of follicular cells with moderate nuclear atypia (original magnification, ×10; Papanicolaou staining). (*B3*) CNB showing neoplastic cells arranged in solid and trabecular architecture (original magnifications, ×4 and ×10; H&E staining). (*B4*) Immunohistochemical staining showing focal weak immunoreactivity for thyroglobulin and diffuse immunoreactivity for TTF-1 and PAX8. **(C)** Representative cytological and histological images of a case of TL. (*C1*) CT scan with i.v. contrast imaging. (*C2*) FNAC sample not diagnostic for the presence of extensive crush artifacts (original magnification, ×20; Papanicolaou staining). (*C3*) CNB provided a fragment of tissue composed of muscular tissue with intense lymphoid infiltration (original magnifications, ×4 and ×10; H&E staining). (*C4*) Immunohistochemical staining showing that neoplastic cells were CD20 positive and CD3 negative with high proliferative indices compatible with B-cell lymphoma. **(D)** Representative cytological and histological images of a case of carcinoma of extra-thyroid origin. (*D1*) CT scan with i.v contrast imaging. (*D2*) FNAC sample showing a few groups of epithelial cells with marked nuclear atypia (original magnifications, ×10 and ×40 in the *insert*; Papanicolaou staining). (*D3*) CNB showing a few clusters of neoplastic cells interspersed in fibrotic stroma (original magnifications, ×4 and ×10; H&E staining). (*D4*) Immunohistochemical staining showing the absence of immunoreactivity for thyroglobulin, TTF-1, and PAX8, suggesting an extra-thyroid origin.

The correlation of the results of CT scan with those of FNAC, CNB, and specific immunohistochemical staining for ATC, PDTC, TL, and TGM of lung adenocarcinoma is shown in **Figure 1**.

### FNAC and CNB Results

We firstly analyzed the ability of both FNA and CNB to provide adequate tumor samples to reach a diagnosis. Enough and adequate material to perform the cytological analysis was obtained in 76 out of 95 (80%) FNA specimens. A significantly higher percentage of good quality tissue, defined as a minimum 20% of tumor content in the specimens, was obtained with CNB (92/95, 96.8%; *p* < 0.01). Using this material, immunohistochemistry was performed in 89 of 95 (93.7%) cases, which was useful to the pathologists in clarifying the diagnosis in 88 of 89 (98.9%) cases. The main immunohistochemical markers evaluated according to the histotype revealed by CNB are reported in **Table 2**. The molecular analysis performed in 17 cases is reported in **Table 3**.

**Table 2:** Panel of the main immunohistochemical markers evaluated in our series of rapidly growing thyroid masses according to histology diagnosed by CNB (*n* = 89).

		ATC^a^ (*n* = 33)	PDTC^a^ (*n* = 26)	TL (*n* = 12)	SCC^b^ (*n* = 7)	TGM (*n* = 6)	Other cancers (*n* = 5)
Pan-cytokeratins	+++	3 (9.1%)	4 (15.4%)	–	1 (14.3%)	2 (33.3%)	1 (20%)
	+/−	1 (3%)	–	–	–	1 (16.7%)	–
	Negative	10 (30.3%)	–	4 (33.3%)	1 (14.3%)	–	1 (20%)
	Not performed	19 (57.6%)	22 (84.6%)	8 (66.7%)	5 (71.4%)	3 (50%)	3 (60%)
Cytokeratin CAM 5.2	+++	12 (36.4%)	7 (26.9%)	–	3 (42.8%)	2 (33.3%)	–
	+/−	11 (33.3%)	1 (3.8%)	–	–	1 (16.7%)	–
	Negative	8 (24.2%)	1 (3.8%)	2 (16.7%)	–	1 (16.7%)	4 (80%)
	Not performed	2 (6.1%)	17 (65.4%)	10 (83.3%)	4 (57.2%)	2 (33.3%)	1 (20%)
Tg	+++	–	5 (15.2%)	–	–	–	–
	+/−	–	5 (15.2%)	–	–	–	–
	Negative	32 (97%)	16 (61.5%)	2 (16.7%)	7 (100%)	6 (100%)	5 (100%)
	Not performed	1 (3%)	–	10 (83.3%)	–	–	–
TTF-1	+++	1 (3%)	12 (46.2%)	–	–	–	–
	+/−	1 (3%)	2 (7.7%)	–	–	–	–
	Negative	30 (91%)	11 (42.3%)	1 (8.3%)	7 (100%)	6 (100%)	5 (100%)
	Not performed	1 (3%)	1 (3.8%)	11 (91.7%)	–	–	–
PAX-8	+++	3 (9.1%)	8 (30.8%)	–	–	–	–
	+/−	–	1 (3.8%)	–	2 (28.6%)	–	–
	Negative	7 (21.2%)	3 (11.5%)	–	–	2 (33.3%)	3 (60%)
	Not performed	23 (69.7%)	14 (53.8%)	12 (100%)	5 (71.4%)	4 (66.7%)	2 (40%)
PAX-5	+++	–	–	3 (25%)	–	–	–
	+/−	–	–	3 (25%)	2 (28.6%)	–	–
	Negative	–	–	–	–	–	–
	Not performed	33 (100%)	26 (100%)	6 (50%)	5 (71.4%)	6 (100%)	5 (80%)
Ki-67	>30%	7 (21.2%)	2 (7.7%)	7 (58.3%)	1 (14.3%)	–	1 (20%)
	5–30%	–	–	–	–	–	1 (20%)
	<5%	–	–	–	–	–	1 (20%)
	Not performed	26 (78.8%)	24 (92.3%)	5 (41.7%)	6 (85.6%)	6 (100%)	2 (40%)
CD45, other lymphoid markers	+++	–	–	12 (100%)	–	–	–
	+/−	–	–	–	–	–	–
	Negative	8 (24.2%)	1 (3.8%)	–	–	1 (16.7%)	–
	Not performed	25 (75.8%)	25 (96.2%)	–	7 (100%)	5 (83.3%)	5 (100%)

Other cancers include: angiosarcoma (n = 2), undifferentiated mesenchymal neoplasia (n = 2), and plasmacytoma (n = 1). +++ indicates positive staining; +/− indicates focal positive staining; and − indicates negative staining.

CNB, core needle biopsy; ATC, anaplastic thyroid cancer; PDTC, poorly differentiated thyroid cancer; TL, thyroid lymphoma; SCC, squamous cell carcinoma; TGM, thyroid gland metastasis; TTF-1, thyroid transcription factor 1.

a In two ATC cases and one PDTC case, immunohistochemistry was not performed.

b In SCC, positivity for p40 and p63 was demonstrated in all CNB procedures.

**Table 3:** Results of the molecular analysis of 17 cases.

Histotype	Molecular analysis results^a^
ATC	*BRAF*, *H-RAS*, *K-RAS*, and *N-RAS* negative
ATC	*BRAF*, *H-RAS*, *K-RAS*, and *N-RAS*, and *RET/PTC* negative
ATC	*N-RAS* positive; *BRAF*, *H-RAS*, *K-RAS*, and *RET/PTC* negative
ATC	*N-RAS* positive; *BRAF*, *H-RAS*, *K-RAS*, *RET/PTC*, and *TERT* negative
ATC	*BRAF*, *H-RAS*, *H-RAS*, and *N-RAS* negative
ATC	*BRAF*, *H-RAS*, *K-RAS*, and *N-RAS* negative
PDTC	*BRAF*, *H-RAS*, *K-RAS*, *N-RAS*, *EGFR*, *HER-2*, and *PIK3CA* negative
PDTC	*NTRK* fusion (40% of the analyzed cells) positive; *BRAF*, *H-RAS*, *K-RAS*, *N-RAS*, and *TERT* negative
PDTC	*K-RAS* positive; *EGFR* negative
PDTC	*BRAF*, *H-RAS*, *K-RAS*, and *N-RAS* negative
PDTC	*BRAF*, *H-RAS*, *K-RAS*, *N-RAS*, *NTRK1/2/3*, *RET/PTC*, *PAX8*/*PPAR* gamma, and *TP53* negative
PDTC	*RET/PTC*, *ROS1*, and *EGFR* negative
PDTC	*BRAF*, *K-RAS*, and *EGFR* negative
PDTC	*BRAF* positive
TGM from colon carcinoma	*BRAF*, *H-RAS*, and *N-RAS* negative
TGM from lung carcinoma	*BRAF*, *K-RAS*, *EGFR*, *RET*, and *ROS-1* negative
TL	*MYC* translocation positive; *BCL* translocation negative

ATC, anaplastic thyroid carcinoma; PDTC, poorly differentiated thyroid carcinoma; TGM, thyroid gland metastasis; TL, thyroid lymphoma.

a Specific molecular profiling was performed according to the histotype.

A comparison of the cytological results of FNAC and the histological results of CNB is reported in **Table 4**. FNAC was able to identify a malignant lesion in 74 of 95 (77.9%) cases: in 12 out of 74 (16.2%), it provided suspicion for malignancy (TIR 4); in 54 of 74 (73%), it was definitively positive for malignancy (TIR 5); and in 8 of 74 (10.8%) cases, it was suggestive for TL. In 19 (20%) cases, FNAC was not diagnostic (TIR 1), likely due to the presence of necrotic material and inflammatory cells not clearly identifiable during the neck US; in two cases (2.1%), an indeterminate cytology was obtained (TIR 3).

**Table 4:** Comparison of the results of FNAC and CNB in rapidly growing thyroid masses (*n* = 95).

			CNB						Total
			ATC	PDTC	TL	TGM^a^	Other Cancers^b^	Not Diagnostic	
**FNAC**	TIR 1	Not diagnostic	5	4	5	3	2	–	19 (20%)
	TIR 2	Benign	–	–	–	–	–	–	–
	TIR 3	Indeterminate	1	–	–	–	1	–	2 (2.1%)
	TIR 4	Suspicious carcinoma	4	4	–	1	2	1	12 (12.6%)
	TIR 5	ATC	6	–	–	–			55 (57.9%)
		Malignant neoplasia	19	19	1	2	5	1	
		Squamous cell carcinoma	–	–	–	–	2		
	TL		–	–	6	–	–	1	7 (7.4%)
Total			35 (36.8%)	27 (28.4%)	12 (12.6%)	6 (6.3%)	12 (12.6%)	3 (3.2%)	95 (100%)

FNAC, fine needle aspiration cytology; CNB, core needle biopsy; ATC, anaplastic thyroid carcinoma; PDTC, poorly differentiated thyroid carcinoma; TL, thyroid lymphoma; TGM, thyroid gland metastases.

a Kidney, colon, lung (n = 2) and breast (n = 2).

b Angiosarcoma (n = 2), undifferentiated mesenchymal neoplasia (n = 2), squamous cell carcinoma (n = 7), and plasmacytoma (n = 1).

**Figure 2:**
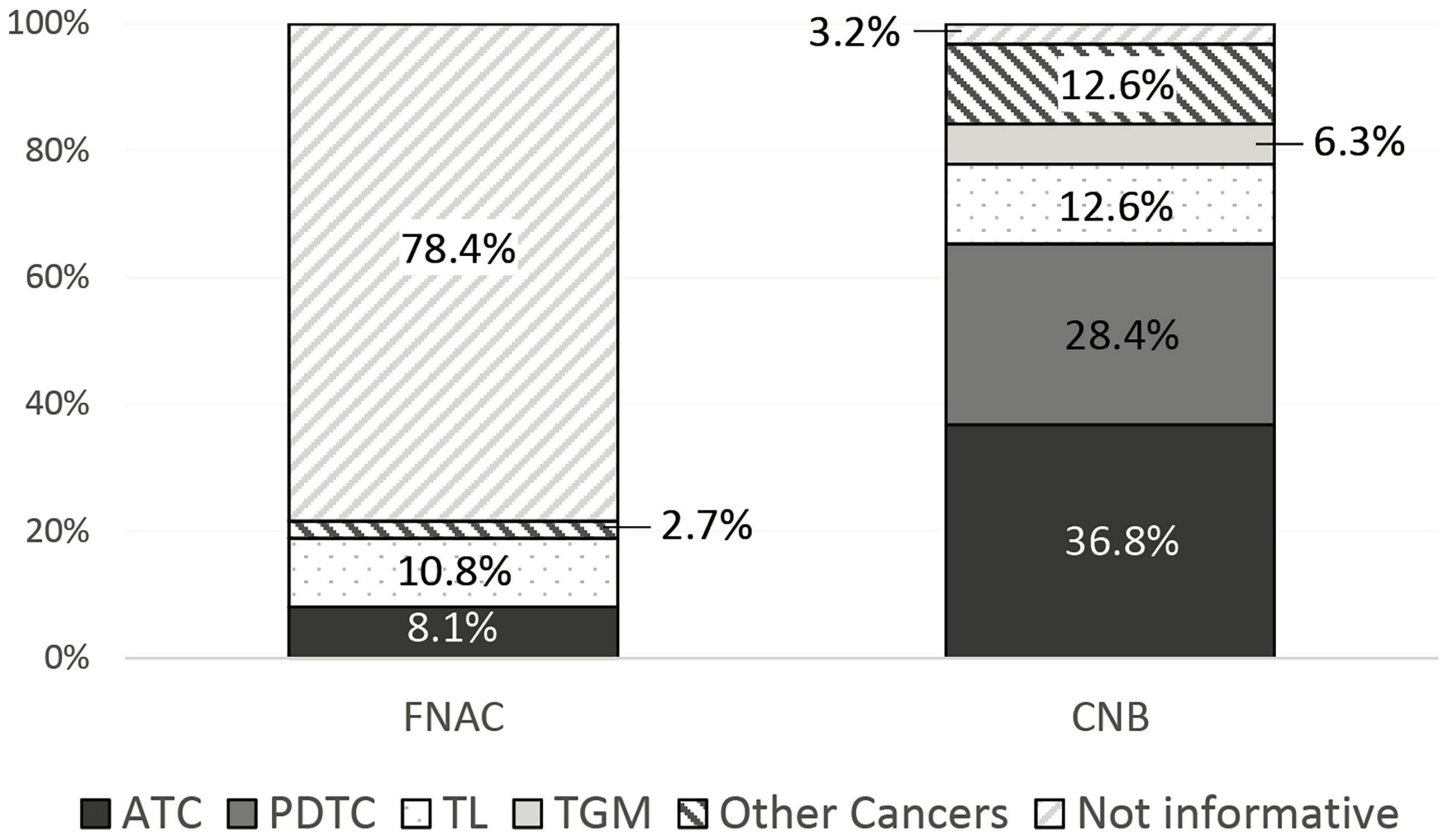
Diagnostic ability of fine needle aspiration cytology (FNAC) and core needle biopsy (CNB) in identifying tumor histotype.

Conversely, CNB was able to diagnose the malignant lesion in all but three cases (92/95, 96.8%). As expected, in all diagnostic specimens, CNB was able to define the histotype of the malignancy, while FNAC did it in 16 of 74 (21.6%) cases (*p* < 0.01) (**Figure 2**).

ATC was diagnosed overall in 35 out of 95 (36.8%) cases by CNB. Conversely, FNAC was suspicious for or diagnostic of malignancy in most of the ATCs identified by CNB (29/35, 82.9%), but the specific diagnosis of ATC was only made in six of them (17.1%).

Similarly, FNAC was suspicious for or diagnostic of malignancy in 23 of 27 (85.2%) PDTCs identified by CNB, but the specific diagnosis of PDTC could not be established in any of them based on the cytological specimen.

Of the 13 cases of TL, 12 were correctly identified by CNB (92.3%). On the other hand, FNAC provided the correct diagnosis of TL in seven cases (53.8%), and another one (7.7%) was classified as thyroid malignancy (TIR 5), but it was not diagnostic in the other five (38.4%).

Six cases in the whole series had TGM (6.3%), and CNB showed that they were metastases from the kidney, colon, lung (two cases), and breast (two cases). Conversely, FNAC was inconclusive (TIR 1) in three cases, suspicious for malignancy in one case, and positive for malignancy in two cases, but in all of them, FNAC did not perform a correct histological diagnosis, only predicted it correctly.

Moreover, several other rare neck cancers (12/95, 12.6%) were diagnosed in our series (**Table 4**). In 9 out of 12 cases, FNAC was able to identify the malignancy of the lesion and, in two of them, also suggested the presence of squamous cell carcinoma. Conversely, in all cases of rare neck cancers, CNB correctly identified both the malignancy of the lesion and the histotype.

### Comparison of the Results of CNB, FNAC, and Histology

In 24 of 95 (25.2%) patients, surgery was performed because there was no preoperative evidence of cervical bundle and massive esophageal and/or tracheal infiltration, as assessed by CT scan, bronchoscopy, and esophagoscopy. In this subgroup, we compared the histology and the results of both CNB and FNAC.

As shown in **Table 5**, in 20 out of 24 (83.3%) cases, the CNB results were concordant with those of histology, while in 3 of 24 (12.5%) cases, CNB showed a PDTC, but the histology demonstrated the presence of ATC. In only one case of those treated with surgery was the material obtained by CNB inadequate to reach a specific histological diagnosis.

**Table 5:** Diagnostic performance of FNAC and CNB compared with histology in patients treated with surgery (*n* = 24).

Patient	Age (years)	Sex	FNAC	FNAC histotype definition	CNB	CNB histotype definition	Histology
1	63	F	TIR 3	No	Undifferentiated mesenchymal neoplasia	Yes	Undifferentiated mesenchymal neoplasia
2	63	M	TIR 4	No	SCC	Yes	SCC
3	57	F	TIR 5	No	PDTC	Yes	PDTC
4	60	M	TIR 4	No	PDTC	Yes	PDTC
5	70	F	TIR 5	No	PDTC	Yes	PDTC
6	68	M	TIR 5	No	PDTC	Yes	PDTC
7	56	F	TIR 5	No	PDTC	Yes	PDTC
8	33	M	TIR 5	No	PDTC	Yes	PDTC
9	70	M	TIR 5	No	PDTC	Yes	PDTC
10	62	M	TIR 5	No	PDTC	Yes	PDTC
11	45	M	TIR 5	**Yes**	PDTC	Yes	PDTC
12	40	M	TIR 4	**Yes**	Not diagnostic	No	PDTC
13	82	M	TIR 1	No	PDTC	Yes	ATC
14	70	F	TIR 4	No	PDTC	Yes	ATC
15	53	F	TIR 4	No	PDTC	Yes	ATC
16	78	M	TIR 1	No	ATC	Yes	ATC
17	68	M	TIR 4	No	ATC	Yes	ATC
18	62	F	TIR 1	No	ATC	Yes	ATC
19	52	F	TIR 5	No	ATC	Yes	ATC
20	72	F	TIR 5	**Yes**	ATC	Yes	ATC
21	63	F	TIR 4	No	ATC	Yes	ATC
22	58	F	TIR 5	No	ATC	Yes	ATC
23	91	F	TIR 5	No	ATC	Yes	ATC
24	47	F	TIR 5	No	ATC	Yes	ATC

FNAC, fine needle aspiration cytology; CNB, core needle biopsy; ATC, anaplastic thyroid carcinoma; PDTC, poorly differentiated thyroid carcinoma; SCC, squamous cell carcinoma.

Regarding FNAC, the cytological material was inadequate to reach a diagnosis in three cases (TIR 1), in one case showed an indeterminate lesion (TIR 3), and in 20 out of 24 (83.3%) cases was suggestive or suspicious for malignancy not further characterized. Overall, the ability of FNAC to define the histotype concordantly with histology was observed in only three cases (12.5%) (**Table 5**).

### Complications Related to FNAC or CNB

In our series, no complications related to FNAC and CNB were experienced during and after the procedures.

## Discussion

Large thyroid masses, particularly if rapidly growing, represent a clinical challenge as they are often related to life-threatening events. In order to quickly reach a diagnosis and carry out appropriate treatments, several procedures have been proposed (3, 25). Surgical conventional biopsy achieves diagnosis in almost all cases, but it has several limitations. It may increase the morbidity and mortality, especially in the elderly, is time-consuming, is invasive, may cause tissue damage, and often requires hospital admission, general anesthesia, and potential transfer to the intensive care unit. Therefore, due to the surgical risk, this approach is not be suitable for all patients (26). Conversely, FNAC is usually the first type of biopsy chosen because it is more immediate, is performed without general anesthesia, and can allow the diagnosis of malignancy in >60% of ATC cases (27, 28). However, cytology, particularly in rare and high-grade malignant neoplasms, does not often give information about the histotype of the tumor, which is, indeed, necessary for the planning of further therapeutic procedures (29). Because of the presence of necrotic material and inflammatory cells in aspirates, we also had 20% of non-diagnostic cases, a higher prevalence when compared to the non-diagnostic FNAC commonly observed when thyroid nodules were submitted to the procedure in our center (30). This percentage of non-diagnostic cases could potentially be reduced by using cell block specimens on the FNA material. However, this is not a routine procedure, the rate of non-diagnostic results could be slightly reduced (31), and, although commonly applied in our center in lung and liver lesions, it is not performed on thyroid nodules.

Several types of neoplasia were diagnosed in our series. Indeed, beyond ATC and PDTC, the most frequently diagnosed neoplasia on CNB (36.8% and 28.4%, respectively), TL (13.7%), TGM (6.3%), and other rare neck cancers (12.6%), such as angiosarcoma, mesenchymal neoplasia, squamous cell carcinoma, and plasmacytoma, were also diagnosed. Similar results were reported by Choi et al. (32), who performed FNAC and CNB in a series of 52 thyroid nodules clinically suspicious for TGM. Unlike in our study, FNAC and CNB were not performed in all cases; nevertheless, their study demonstrated the higher sensitivity of CNB *vs*. FNAC in the diagnosis of TGM.

It was clearly demonstrated that 18.9% (18/95) of these masses, although localized in the thyroid, were indeed either metastases from other types of tumors or quite rare tumors. However, none of them was identified by FNAC, but were clearly recognized by CNB, which is a true biopsy and allows the pathologist to perform immunohistochemistry, which can better characterize the origin of the tumor (33).

The role of CNB compared to FNAC has already been explored in the diagnosis of thyroid nodules, particularly in cytologically indeterminate lesions. Na et al. (34) evaluated 161 patients with indeterminate cytology and confirmed the higher sensitivity of CNB compared to FNAC in detecting malignancy. However, in our experience, the CNB procedure required that the nodule dimension be ≥3 cm, thus representing a limitation in its routine application. The higher sensitivity of CNB compared to FNAC in detecting malignancy was also confirmed in other human tumors (35–38).

Regarding large neck masses, in particular ATC, Ha et al. (26) evaluated the diagnostic performance of FNAC and CNB in a series of patients with ATC or TL. However, FNAC was performed in 83.8% and CNB in only 32.2% of the study group. The authors obtained 100% and 90.9% positive predictive values for CNB and FNAC in diagnosing ATC, respectively. In our series, the positive predictive values for ATC diagnosis were 100% and 17.1% and for TL diagnosis were 92.3% and 61.5% for CNB and FNAC, respectively. Moreover, unlike in our study, their data were retrospectively collected, CNB was performed in only a minority of cases, and only in a few patients (8.1%) were CNB and FNAC simultaneously performed.

In our series, in the 24 cases submitted to surgery, the correlation between the CNB and histology results was very high since in only 12% of the cases was it slightly discordant. This could be, at least in part, due to the heterogeneity of the ATC, being either of pure anaplastic origin or deriving from the dedifferentiation of a preexisting PTC (3). In any case, this discordance did not play any role in the management of the patients. In the era of precision medicine, in which treatments are targeted against specific genes and mutations of the tumor, the ability to perform a rapid and correct histological diagnosis cannot be overlooked. Indeed, particularly in ATC cases, in which a definitive cure is unlikely with standard treatments, the molecular signature of the neoplasia could improve the outcome of patients harboring actionable mutations (i.e., *BRAF* V600E) (24, 39), as well as in a neoadjuvant setting (40). In our series, although the material obtained from CNB was optimal to perform molecular analysis in most cases (96.8%), it was performed in only 17.9%. This quite low frequency of analyzed cases is not unexpected since the knowledge about the impact of molecular analysis on the treatment of patients with ATC is quite recent (41). Molecular analysis could also be performed on cytological material (42); however, in several cases, cytology does not provide correct information on the histotype, limiting the choice of genetic profile to be analyzed. Conversely, when the histology is known, as in most cases of CNB, a specific molecular profile can be studied, being different not only according to different tumors (43) but also in the context of different thyroid tumors (44, 45). Accordingly, in metastatic malignancies for which the primary site is unknown, the key role of CNB compared to FNAC has been clearly demonstrated, both in clarifying the primary site of the tumor and in obtaining sufficient material to perform the molecular analysis (8, 46).

CNB is a safe and well-tolerated procedure associated with a low incidence of complications when performed in expert hands (47). However, several potential complications have been reported, such as hematoma, voice change, infection, edema, vasovagal reaction, hemoptysis, and dysphagia (34, 48, 49). In a large single-center study, in which CNB was performed on 6,687 thyroid nodules, very few major (0.06%) and minor (0.79%) complications were described (50). Also, in other studies, low rates of major complications, such as bleeding and tumor cell seeding, were observed (51).

These findings are in accordance with our experience, although in the different setting of large thyroid masses, we did not experience any complications related to the CNB procedure. However, to minimize complications, CNB should be performed by well-trained physicians, under real-time US guidance and with a good awareness of neck anatomy and potential complications (52).

To our knowledge, this is the first prospective study comparing the diagnostic performance of FNAC and CNB in a large series of rapidly growing thyroid masses. The main limitation of our study was that it was performed in a tertiary referral center for the treatment of thyroid cancer, therefore making our results not completely reproducible in routine clinical practice. However, it is recommended that these rare thyroid masses should be managed in referral centers able to perform these kinds of procedures. Conversely, the strengths of this study included the large number of patients enrolled; the use of neck US to identify the areas suitable for biopsy, avoiding the presence of tissue necrosis and inflammation; and the simultaneous use of both FNAC and CNB on the same patient, at the same time, from the same skin incision.

## Conclusions

In conclusion, our study demonstrates that CNB is a safe procedure able to optimize the diagnostic times and to obtain an early histological diagnosis, which is fundamental to starting an early and specific treatment. Moreover, the CNB sample can also be immediately analyzed for its molecular profile, with the great advantage that, if a druggable mutation is revealed, a more specific and active drug can be immediately started. This evidence strongly supports the official introduction of CNB in routine clinical practice for the diagnosis of large and rapidly growing thyroid masses.

## Data Availability Statement

The original contributions presented in the study are included in the article/Supplementary Material. Further inquiries can be directed to the corresponding author.

## Ethics Statement

The studies involving human participants were reviewed and approved by Comitato Etico Area Vasta Nord Ovest (CEAVNO). The patients/participants provided written informed consent to participate in this study.

## Author Contributions

AM, LDN, RE, and GM: conceptualization, methodology and data curation. LDN, AA, PP, CEA, and GM: surgical procedures. RC: imaging revision. LT, CU, and FB: cytological and histological analysis. AM and LDN: formal analysis. AM, LDN, AA, PP, CEA, EM, RE, and GM: investigation. AM, LDN, and RE: writing—original draft preparation. All authors: writing—review and editing. RE and GM: supervision. All authors contributed to the article and have read and approved the submitted version.

## Funding

The work was supported by the Ministero dell’Istruzione, dell’Università e della Ricerca Italiano (MIUR, investigator grant 2017, project code PRIN 2017YTWKWH).

## Conflict of Interest

The authors declare that the research was conducted in the absence of any commercial or financial relationships that could be construed as a potential conflict of interest.

## Publisher’s Note

All claims expressed in this article are solely those of the authors and do not necessarily represent those of their affiliated organizations, or those of the publisher, the editors and the reviewers. Any product that may be evaluated in this article, or claim that may be made by its manufacturer, is not guaranteed or endorsed by the publisher.
